# Comparative Genomics and Transcriptional Analysis of *Flavobacterium columnare* Strain ATCC 49512

**DOI:** 10.3389/fmicb.2017.00588

**Published:** 2017-04-19

**Authors:** Hasan C. Tekedar, Attila Karsi, Joseph S. Reddy, Seong W. Nho, Safak Kalindamar, Mark L. Lawrence

**Affiliations:** ^1^College of Veterinary Medicine, Mississippi State UniversityMississippi State, MS, USA; ^2^Mayo Clinic, Department of Health Sciences ResearchJacksonville, FL, USA

**Keywords:** *Flavobacterium columnare*, comparative genomics analysis, fish health, denitrification, RNA-Seq

## Abstract

*Flavobacterium columnare* is a Gram-negative fish pathogen causing columnaris disease in wild and cultured fish species. Although the pathogen is widespread in aquatic environments and fish worldwide, little is known about biology of *F. columnare* and mechanisms of columnaris disease pathogenesis. Previously we presented the complete genome sequence of *F. columnare* strain ATCC 49512. Here we present a comparison of the strain ATCC 49512 genome to four other *Flavobacterium* genomes. In this analysis, we identified predicted proteins whose functions indicate *F. columnare* is capable of denitrification, which would enable anaerobic growth in aquatic pond sediments. Anaerobic growth of *F. columnare* ATCC 49512 with nitrate supplementation was detected experimentally. *F. columnare* ATCC 49512 had a relatively high number of insertion sequences and genomic islands compared to the other *Flavobacterium* species, suggesting a larger degree of horizontal gene exchange and genome plasticity. A type VI subtype III secretion system was encoded in *F. columnare* along with *F. johnsoniae* and *F. branchiophilum*. RNA sequencing proved to be a valuable technique to improve annotation quality; 41 novel protein coding regions were identified, 16 of which had a non-traditional start site (TTG, GTG, and CTT). Candidate small noncoding RNAs were also identified. Our results improve our understanding of *F. columnare* ATCC 49512 biology, and our results support the use of RNA sequencing to improve annotation of bacterial genomes, particularly for type strains.

## Introduction

*Flavobacterium columnare* is in the family *Flavobacteriaceae*, which is one as the main phyletic lines within the Bacteroidetes (Bernardet et al., 1996). This family has several important fish-pathogens, including *Flavobacterium psychrophilum, Flavobacterium branchiophilum*, and *Flavobacterium columnare*. *F. columnare* is the causative agent of columnaris disease, which is common in freshwater fish throughout the world, infecting populations of wild and cultured fish species. In the United States, channel catfish is the leading aquacultured fish species, and columnaris disease is the second leading cause of mortalities in commercial catfish aquaculture (Durborow et al., 1998).

*F. columnare* is a long Gram-negative rod (2–10 μm in length) forming yellow-pigmented and typically rhizoid colonies (Bernardet et al., 1996). Four distinct colony types have been reported for *F. columnare* strains (Kunttu et al., 2009), which are divided into three genomovars based on 16S rRNA gene-based RFLP (Wakabayashi and Wakabayashi, 1999), and *F. columnare* ATCC 49512 belongs to genomovar I (Michel et al., 2002). Strains from the different genomovars affect geographically distributed fish species at different virulence levels (Arias et al., 2004; Darwish and Ismaiel, 2005; Shoemaker et al., 2008), Genomovar II strains tend to be more virulent than genomovar I strains in most fish species (Olivares-Fuster et al., 2011; Lafrentz et al., 2012), but there is considerable strain variation in virulence within the genomovars. Recently, a standard and optimized protocol was developed to distinguish *F. columnare* isolates using expected restriction patterns for genomovars I, II, II-B, and III (Lafrentz et al., 2014, 2016).

Despite its importance, there is a lack of essential information concerning columnaris disease, which limits the implementation of methods to manage, treat, and prevent the disease. Moreover, mechanisms of *F. columnare* pathogenesis are not completely understood. The nucleotide sequence of a pathogen's genome is a major step for understanding the mechanisms of pathogenesis.

To date, several complete genomes from *Flavobacterium* species have been released, including *F. psychrophilum* (Duchaud et al., 2007), *F. johnsoniae* (McBride et al., 2009), *F. branchiophilum* (Touchon et al., 2011), *F. indicum* (Barbier et al., 2012), and *F. columnare* (Tekedar et al., 2012). Here we present comparative genome analysis of the *F. columnare* ATCC 49512 genome. For the purposes of this comparison, we chose representative closed genomes from five of the best characterized species to enable a more complete functional comparison. The analysis showed, for the first time, that *F. columnare* is capable of denitrification. Transcriptome analysis of strain ATCC 49512 confirmed predicted gene annotations and identified 41 new protein coding regions, 16 of which have a non-traditional start site (TTG, GTG, and CTT). Thirty-six of the newly identified proteins are conserved hypothetical proteins, of which 30 may be involved in virulence based on similarity to proteins in MvirDB (Zhou et al., 2007). Our results provide important new information about the physiology of *F. columnare* and yield strong evidence for the utility of RNA-Seq to improve annotation of bacterial genomes.

## Materials and methods

### Library preparation and sequencing

*F. columnare* strain ATCC 49512 is the type strain in the genomovar I group; it was isolated in 1987 from a skin lesion of brown trout (*Salmo trutta*) fry in France (Bernardet, 1989). Bacteria were cultivated in *Flavobacterium columnare* growth medium (FCGM) broth or agar (Farmer, 2004) at 30°C with shaking at 200 rpm. Genomic DNA was isolated and short, medium, and large insert libraries were prepared and sequenced as described (Tekedar et al., 2012).

### Anaerobic growth

The ability of *F. columnare* ATCC 49512 to grow anaerobically using nitrate as an electron acceptor was determined. Bacteria were grown under aerobic conditions in FCGM broth [tryptone (8.00 g), yeast extract (0.80 g), MgSO_4_ 7 H2O (1.00 g), CaCl_2_ 2H_2_O (0.74 g), NaCl (5.00 g), and sodium citrate (1.50 g) per liter] to an OD_600_ of 1.0. The culture was diluted 1/10 in FCGM and FCGM with 5 mM sodium nitrate broth medium and incubated under anaerobic conditions at 30°C using a GasPak anaerobic system (Fisher Scientific, PA, USA) in a standard anaerobic jar. Bacterial growth was monitored using OD_600_ for 1 week.

### Determination of extracellular nitrite

Nitrite is an intermediate product during denitrification and is reduced to nitric oxide by nitrite reductase. To determine nitrite production during anaerobic growth, a diazotization reaction was used as described (Griess, 1879) with some modifications. Supernatant was collected from cultured bacteria following centrifugation, and 100 μl was transferred to a 96-well plate (Corning, NY, USA). Serially diluted sodium nitrite was used for a standard curve. Fifty microliters of sulfanilamide solution (Sigma-Aldrich; 1% sulfanilamide in 5% phosphoric acid) was added to each sample, and the plate was incubated for 10 min without light. Finally, 50 μl of N-1-naphthylethylenediamine dihydrochloride (NED) solution (Sigma-Aldrich; 0.1% NED in water) was added to each followed by another 10-min incubation without light. Color development was measured at 540 nM using a microplate reader (Molecular Devices, CA, USA).

### Comparative analysis of *Flavobacterium* genomes

Five publicly available annotated *Flavobacterium* genomes (*Flavobacterium columnare* ATCC 49512 [CP003222], *Flavobacterium branchiophilum* FL-15 [NC_016001], *Flavobacterium indicum* GPTSA100-9^T^ [NC_017025], *Flavobacterium johnsoniae* UW101 [NC_009441], and *Flavobacterium psychrophilum* JIP02/86 [AM398681]) were used for genome comparisons. For pathway analyses and comparison of gliding motility components, Kyoto Encyclopedia of Genes and Genomes (KEGG) Pathway Database (http://www.genome.jp/kegg/) was used (Ogata et al., 1999; Kanehisa and Goto, 2000). Rast annotation was used to conduct a sequence-based comparison of the *Flavobacterium* genomes (Overbeek et al., 2014).

All the annotated *Flavobacterium* genome proteins were categorized according to their function using BAsys annotation server (Van Domselaar et al., 2005). Mauve genome alignment software was used to identify potential horizontal transfer loci and genomic rearrangements (Darling et al., 2004). PSORTdb (http://db.psort.org) version 3.0 was used to predict protein localization (Rey et al., 2005; Yu et al., 2011). Orthology analysis was conducted using OrtholugeDB (Whiteside et al., 2013) and Rast annotation server (Aziz et al., 2008; Overbeek et al., 2014).

EDGAR was used to determine phylogenetic relationships between the *Flavobacterium* genomes and to calculate genome to genome distances (Blom et al., 2009). Pan-core genome and singleton analysis of the *Flavobacterium* genomes was conducted with EDGAR (Blom et al., 2009). EDGAR was also used to create a synteny plot for the *Flavobacterium* genomes, which helps to show colocalization of genes on a stretch of DNA (Blom et al., 2009).

### Functional systems analysis of *Flavobacterium* genomes

Genome-wide transcriptional regulatory networks (TRNs) were computationally predicted using P2TF (Predicted Prokaryotic Transcriptional Factors) and P2CS (Prokaryotic 2-Component System) (Ortet et al., 2012, 2015). In these analyses, bacterial transcription factors, one-component systems (OCS), DNA-binding response regulators (RR), sigma factors (SF), transcriptional regulators (TR), other DNA-binding proteins (ODP), two-component systems, histidine kinases (classic, hybrid, and unorthodox), and phosphotransferase proteins [histidine phosphotransferases (Hpt) and histidine kinase homodimeric domain proteins (HisKA)] were computationally predicted.

MacSyFinder and TXSScan were used to detect protein secretion systems and their appendages in the *Flavobacterium* genomes (Abby et al., 2014). Protein files were downloaded from NCBI database for each genome. “Ordered replicon” was used as the type of dataset, and “circular” was used as the topology setting. Default parameters were used for detection of all available secretion systems (maximal *e*-value: 1.0, maximal independent *E*-value: 0.001, minimal profile coverage: 0.5).

CRISPR elements were identified for the five *Flavobacterium* genomes using CRISPRfinder (Grissa et al., 2007). CAS elements were identified using MacSyFinder (Abby et al., 2014). Maximal *E*-value was set at 1.0, maximal independent *E*-value was 0.001, and minimal profile coverage parameters were set for CAS element identification.

To identify possible virulence factors encoded in *F. columnare* ATCC 49512 and other *Flavobacterium* strains, Microbial Virulence Database (MVirDB) was downloaded from http://mvirdb.llnl.gov/ (Zhou et al., 2007), and local BLAST was conducted with all the predicted ATCC 49512 and other *Flavobacterium* strains, proteins using CLC Genomic Workbench (version 6.5). To be considered a match, *Flavobacterium* proteins had to have BLAST result in the MvirDB with an *E*-value <1*10^−10^. For comparison with other *Flavobacterium* strains, the predicted *F. columnare* ATCC 49512 virulence factor proteins were uploaded to the NCBI website to identify the best BLAST hit results. Distribution of predicted *F. columnare* ATCC 49512 virulence factors in metabolic pathways was analyzed using Blast2GO (Conesa et al., 2005).

### Genomics islands, insertion elements, and phage analysis

IslandViewer was used to predict genomic islands in the completed *Flavobacterium* genomes (Langille and Brinkman, 2009; Dhillon et al., 2013). IslandViewer combines three different genomic island prediction methods: IslandPick (Langille et al., 2008), SIGI-HMM (Waack et al., 2006), and IslandPath-DIMOB (Hsiao et al., 2003). Issaga was used to identify individual insertion sequences (Varani et al., 2011).

To identify phage elements in *Flavobacterium* genomes, Genbank files were downloaded for all the evaluated *Flavobacterium* genomes and submitted to the PHASTER (PHAge Search Tool- Enhanced Release) server (Arndt et al., 2016).

### Transcriptome analysis by RNA-Seq

For RNA isolation, *F. columnare* strain ATCC 49512 was grown in 10 ml Shieh broth at 30°C to log phase (OD_600_ 0.8) and harvested by centrifugation. RNAprotect Bacteria Reagent (Qiagen) was immediately added, and pellets were stored at −80°C. Total RNA was isolated using RNA Qiagen Mini Kit, and rRNA was removed with the Ribo-Zero™ Magnetic rRNA removal kit (Illumina, San Diego, CA). Sequencing libraries were prepared using Truseq RNA v2 (Illumina) at Global Biologics (Columbia, MO) and pooled. Single-end 50 bp reads were sequenced on two lanes using an Illumina HiSeq 2000.

To validate genome annotation generated by the NCBI Prokaryotic Genome Annotation Pipeline (08.13.2015) and to identify unannotated functional elements, mRNA expression profiling of *F. columnare* was performed using RNA-seq. Transcriptome profiling of *F. columnare* using Illumina Hi-Seq 2000 generated a total of 68,164,700 single-end reads across six samples. Reads were processed for adaptor removal and filtered for quality using FastX clipper and FastQC, respectively. Reads were aligned to the *F. columnare* ATCC 49512 genome (NC_016510.2) using Bowtie 2 (Langmead and Salzberg, 2012) at default settings.

Bacterial Intergenic Region Analysis Pipeline (BIRAP) (https://github.com/jsreddy82/birap) was used for analysis of expression and evaluation of annotation. BIRAP is a command-line Perl pipeline that integrates expression data with *in silico* annotation to identify expressed annotated as well as unannotated regions. The tool capitalizes on the single nucleotide resolution generated by RNA-seq when reads are initially aligned to the genome using tools such as Bowtie and its subsequent representation as reads per base (RPB) in the pileup output generated by SAMtools (Li et al., 2009). The pileup file generated by SAMtools contains all expressed nucleotides and the total number of reads aligned to each.

BIRAP establishes a basal expression cutoff for each sample as the 10th percentile RPB for all expressed bases in the genome (Wurtzel et al., 2010; Kumar et al., 2012; Reddy et al., 2012). After a baseline for expression is established, RPB is binary transformed to represent one of two states. A value of 1 represents expression above baseline while 0 indicates no expression. Once expression across all samples is binary transformed, a statistical model for binary expression is calculated to determine expression at each nucleotide in the genome.

Normalized and binary transformed RNA-seq data is used for evaluation of annotation. An annotated region is considered as expressed only if at least 60% of all bases spanning the length of the gene are above baseline expression. BIRAP then identifies all expressed intergenic regions (EIRs) and verifies their association with annotated regions. A region of at least 70 bp in length, identified as expressed, and located between two annotated regions is classified as an EIR. If an EIR is located within 10 bp up- or downstream of an annotated region, then the EIR is marked as associated with that annotated region in the result file, which helps evaluate the annotated region's start and stop sites. If EIRs are not in the vicinity of annotated regions, they are marked as unpredicted EIRs. BIRAP evaluates EIRs as potentially being putative non-coding RNAs when promoters and terminators are predicted in appropriate locations. Small non-coding RNAs are known to regulate gene expression and are generally under the influence of a promoter or a rho-independent terminator (Reddy et al., 2012).

EIRs identified by BIRAP were searched against the translated nucleotide non-redundant bacterial database using BlastX (Altschul et al., 1997). EIRs mapping along the full length of closely related orthologs was classified as novel protein coding regions. EIRs homologous to 5′ and 3′ ends of proteins in closely related species were used to evaluate start and stop sites of annotated regions in the *F. columnare* genome. Promoters and rho-independent terminators were predicted using Genome2D (Baerends et al., 2004) and TranstermHP (Kingsford et al., 2007), respectively. EIRs identified as putative non-coding RNAs by BIRAP were searched against BlastX to eliminate false positives, and those that remained were searched against Rfam (Gardner et al., 2011) for sequence conservation across other species.

To provide an overview of gene regulation and operon structures, Database of prOkaryotic OpeRons (DOOR2) (Mao et al., 2014) was used to predict operons using the most recent annotation of *F. columnare* ATCC 49512 generated by NCBI. Operons encoding two or more genes were evaluated for expression using the RNA-seq expression profile with BIRAP.

### Accession numbers

The genome sequence of *F. columnare* ATCC 49512 has been deposited in the DDBJ/EMBL/GenBank database under the following accession number: CP003222, version CP003222.2 (GI: 372863588). RNA-Seq accession numbers are: 116-SRR1122816, 117-SRR1122817, 118-SRR1122818, 119-SRR1122819, 120-SRR1122820, 121-SRR1122821.

## Results

### Genome features and comparative genomics

**Figure 1:**
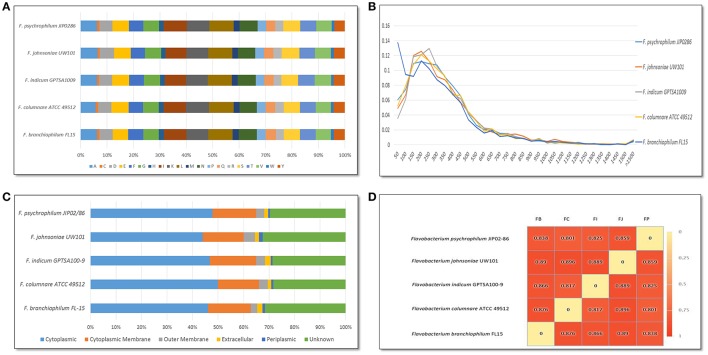
**Comparison of ***Flavobacterium*** genome features. (A)** Amino acid composition for *Flavobacterium* strains. Letter abbreviations: A, Alanine; C, Cysteine; D, Aspartic Acid; E, Glutamic Acid; F, Phenylalanine; G, Glycine; H, Histidine; I, Isoleucine; K, Lysine; L, Leucine; M, Methionine; N, Asparagine; P, Proline; Q, Glutamine; R, Arginine; S, Serine; T, Threonine; V, Valine; W, Tryptophan; Y, Tyrosine. **(B)** Predicted protein lengths for *Flavobacterium* strains. **(C)** Predicted subcellular localization of proteins encoded in *Flavobacterium* genomes using PSORTb. **(D)** Genome to genome distance calculation for *Flavobacterium* genomes. Species abbreviations: FB, *F. branchiophilum* FL15; FC, *F. columnare* ATCC 49512; FI, *F. indicum* GPTSA100-9; FJ, *F. johnsoniae* UW101; and FP, *F. psychrophilum* JIB02-86.

*F. columnare* ATCC 49512 chromosome features compared against *F. johnsoniae* UW101, *F. psychrophilum* JIP02/86, *F. branchiophilum* FL-15, and *F. indicum* GPSTA100-9T are listed in Table 1. All of the *Flavobacterium* strains have low G+C content. Genome size varies from 2,861,988 to 6,096,872 bp, with the *F. johnsoniae* UW101 genome being twice the size of the other flavobacteria. Only *F. psychrophilum* JIP/86 and *F. branchiophilum* FL-15 carry plasmids. Amino acid composition was similar between all the *Flavobacterium* genomes (Figure 1A). Predicted protein lengths were similar for the *Flavobacterium* genomes, except *F. branchiophilum* FL-15 genome had shorter protein lengths than the other four (Figure 1B). Predicted subcellular localization of proteins encoded by the *Flavobacterium* genomes was also similar (Figure 1C). Genome to genome distance calculation showed a better correlation with 16s rRNA gene distances than other evaluation methods (Auch et al., 2010); therefore, we applied this calculation method. Results showed that the *F. columnare* and *F. psychrophilum* genomes are closer than the other *Flavobacterium* genomes (Figure 1D).

**Table 1:** **Chromosome features of ***flavobacterium*** genomes**.

**Chromosome feature**	***F. columnare* ATCC 49512**	***F. psychrophilum* JIP02/86**	***F. branchiophilum* FL-15**	***F. johnsoniae* UW101**	***F. indicum* GPSTA100-9T**
Genome size	3,162,432	2,861,988	3,559,884	6,096,872	2,993,089
G+C content (%)	31.5	32.5	32.9	34.1	31.4
Number of rRNA operons	5	6	3	6	4
Number of tRNA genes	74	49	44	62	55
Protein coding genes	2,642	2,432	2,867	5,056	2,671
Average gene length (bp)	1,021	1,003	1,030	1,061	Unknown
Plasmid	No	Yes	Yes	No	No

### Pan-core and singletons genome analysis

**Figure 2:**
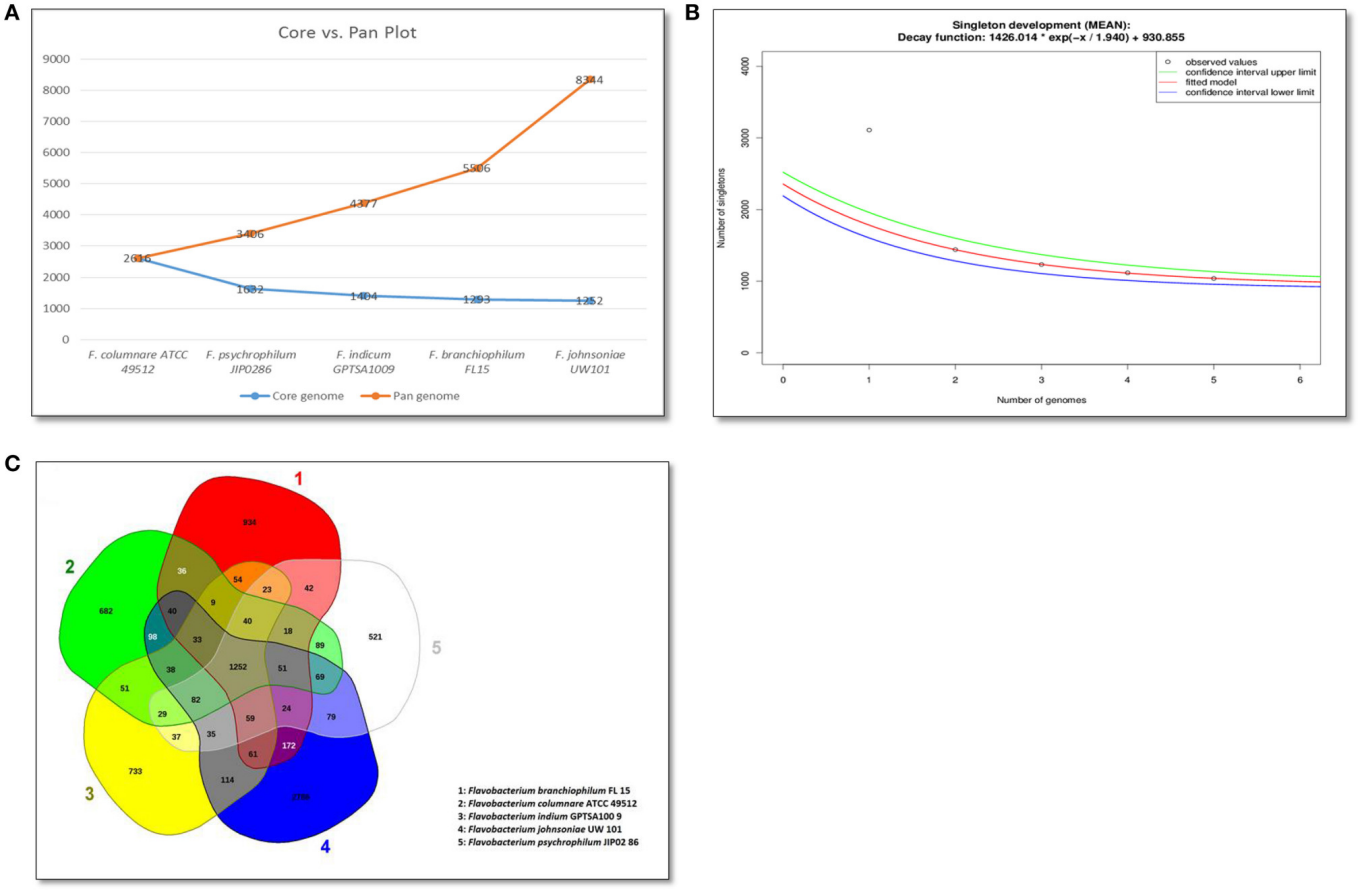
*****Flavobacterium*** pan core genomes analysis**. **(A)** Core vs. pan-genome plot of the five evaluated *Flavobacterium* genomes. **(B)** Singleton (unique genes) analyses for *Flavobacterium* genomes. A decreasing number of singletons are observed with each sequential addition of a genome. **(C)** Venn diagram of *Flavobacterium* pan and core genomes based on orthology analysis.

Bacterial pan-core and singletons analysis are used to identify unique proteins and to estimate the genomic diversity of evaluated genomes (Vernikos et al., 2015). Singletons are defined as genes encoding proteins unique to a particular species in the analysis. Our comparative pan-genome analyses of the five *Flavobacterium* genomes revealed that each *Flavobacterium* species' contributed unique proteins to the pan-genome (Figure 2A). The final core genome size was 1,252 genes, and the final pan-genome had 8,339 genes. An exponential decay model estimated that 930 new genes could be revealed for every new *Flavobacterium* genome added to the analysis (Figure 2B). A pan-core genome Venn diagram showed that *F. columnare* and *F. psychrophilum* had the least number of unique genes, possibly because these two species are the closest related (Figure 2C). *F. branchiophilum* and *F. johnsoniae* had the most shared genes because they are the two largest genomes in the comparison. As expected, *F. johnsoniae* had the highest number of unique genes, followed by *F. branchiophilum*.

Most of the unique genes for each species consisted of hypothetical proteins, insertion elements, phage components, and two component systems. More specifically, the *F. columnare* genome has unique nitrate/nitrite reductase components, IS3/IS911 insertion elements, cobalamin biosynthesis proteins, and CRISPR elements. The *F. branchiophilum* genome has unique IS595 insertion elements, bleomycin resistance proteins, and two-component system proteins. The *F. psychrophilum* genome has unique putative cell surface elements and IS3/IS982/IS256/IS1182 family insertion elements. The *F. indicum* genome has unique IS3/IS110 family proteins, and the *F. johnsoniae* genome has many unique hypothetical proteins and two component elements (Supplementary Table 1). Orthology analysis, described below, was used to better define the unique proteins for each species.

### Orthology analysis

**Figure 3:**
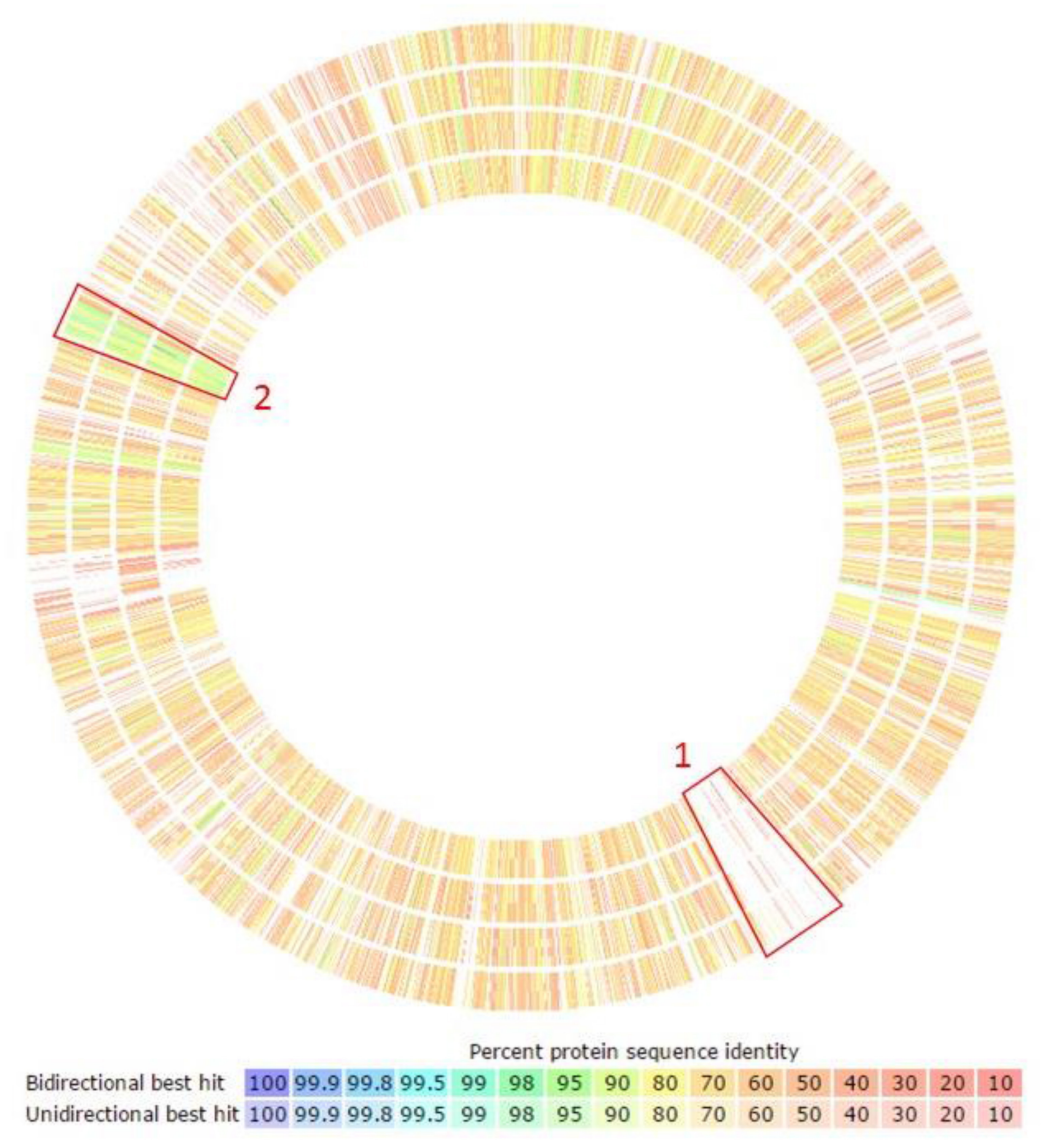
**Orthology analysis of ***F. columnare*** compared against the other ***Flavobacterium*** species using RAST annotation**. Circles display percent identity of proteins from each listed species compared to *F. columnare*. From outside to inside: *Flavobacterium branchiophilum* FL-15, *Flavobacterium indicum* GPTSA100-9, *Flavobacterium johnsoniae* UW101, and *Flavobacterium psychrophilum* JIP02/86. Boxed region 1 indicates a unique *F. columnare* genomic island. Boxed region 2 is conserved in all the evaluated *Flavobacterium* genomes; the genes in this region encode ribosomal subunit elements.

Our orthology analysis clearly identified that the *F. columnare* ATCC 49512 genome has 495 genes encoding unique protein functions compared to the other *Flavobacterium* species analyzed. Of these, 319 encode hypothetical proteins. Many of the protein-coding genes unique to *F. columnare* and many of the conserved protein-coding genes were located in clusters (Figure 3). For example, highlighted region number one shows a unique *F. columnare* genomic island. Highlighted region number two is conserved in all the evaluated *Flavobacterium* genomes; these genes encode ribosomal subunit elements.

One of the protein functions unique to *F. columnare* ATCC 49512 is vitamin B12 synthesis. B12 biosynthesis occurs strictly in some bacteria and archaea; animals and protists require B12 but are not capable of synthesizing it (Martens et al., 2002). In the *F. columnare* ATCC 49512 genome, cobalamin biosynthesis elements, including CbiX, CbiD, CobD, CobW, and CobN subunit were identified. Also, two additional elements were identified that contribute to cobalamin biosynthesis: cobyric acid synthase and a,c-diamide synthase.

The *F. columnare* ATCC 49512 genome also encodes unique nickel permease function, which could play an important role in several cellular processes. Nickel can be essential for energy metabolism, nitrogen metabolism, detoxification, and virulence (Schauer et al., 2007). *Helicobacter pylori* is an example of a species where nickel metabolism contributes to virulence (Atherton, 2006). *F. columnare* ATCC 49512 is also unique in encoding neprilysin, which is considered to have a nutritional role in bacteria. It is a membrane-bound protein that is responsible for the degradation of biologically active peptides (Rawlings and Salvesen, 2013).

*F. branchiophilum* FL-15 encodes 722 unique proteins. Of these, 529 of them are hypothetical proteins. Some of the other genes encode bleomycin resistance proteins, glycoside family proteins, LytTR family two-component system response regulatory proteins, and transposases. Two of the unique proteins in the *F. branchiophilum* genome, TerZ/TerD, are widely present in prokaryotes. They can mediate resistance to tellurite and may be activated by signal transduction pathways triggered by oxidizing agents such as tellurite or hydrogen peroxide. It has been proposed that TerZ/TerD are responsible for intracellular Ca^2+^ signaling (Pan et al., 2011).

A total of 534 proteins is uniquely encoded by *F. indicum* GPTSA100-9. Of these, 424 are hypothetical proteins. Some of the other proteins include a glycosyltransferase, four OmpA outer membrane protein precursors, IS3 and IS110 family transposases, and two-component system response regulatory proteins.

The *F. psychrophilum* JIP0286 genome encodes 360 unique proteins; 293 are annotated as hypothetical proteins. Three two-component system response regulator proteins and a universal stress protein are unique to *F. psychrophilum*. A unique protein encoded by the *bmgB* gene was studied in *Bacteroides fragilis* and shown to play a role in DNA mobilization/horizontal gene transfer (Bass and Hecht, 2002).

As expected, the *F. johnsoniae* UW101 genome has the highest number of unique proteins: 2297. Of these, 1,199 are annotated as hypothetical. Some of these proteins include a ferric citrate transport system: FecR anti-Fecl sigma factor and TonB dependent receptors/siderophores. In this systejom, ferric citrate initiates the signal transduction mechanism by interacting with FecA protein using a TonB-dependent element. The signal is transmitted to FecR, which activates transcription of transport genes (Angerer et al., 1995).

### Transcriptional factors and two-component systems

The percentage of TFs (transcriptional factors) and TCs (two-component systems) in the fish pathogens' genomes [*F. branchiophilum* FL-15 (3.04% TFs, 1.51% TCs), *F. columnare* ATCC49512 (2.49% TFs, 1.17% TCs), and *F. psychrophilum* JIP0286 (2.96% TFs, 1.32% TCs)], is slightly less than the percentage in environmental isolates *F. indicum* GPTSA100-9 (3.11% TFs, 2.28% TCs) and *F. johnsoniae* UW101 (5.59% TFs, 2.89% TCs) (Table 2) (Supplementary Table 2). *F. johnsoniae* UW101 has more TFs and TCs than any other *Flavobacterium* genome, reflecting its more complex environmental niche. Notably, TC response regulator PglZ is conserved among all the *Flavobacterium* genomes. Histidine phosphotransferases (HPT), a part of the TCs, are present only in the *F. branchiophilum* FL-15 and *F. columnare* ATCC49512 genomes. *F. branchiophilum* FL-15 encodes two putative unorthodox histidine kinases. *F. branchiophilum* FL-15 encodes more TFs than the other two fish pathogenic species. However, a similar percentage of histidine kinases and response regulators are encoded in all fish pathogenic *Flavobacterium* genomes.

**Table 2:** **Transcriptional factors and two-component systems in ***Flavobacterium*** genomes**.

	Transcription factors^*^					**Two-component systems**											
						**Histidine kinases**			**Phosphotransfer proteins**		**Response regulators**						
	**OCS**	**RR**	**SF**	**TR**	**ODP**	**Classic**	**Hybrid**	**Unorthodox**	**HPT**	**HisKA**	**OmpR**	**NarL**	**NtrC**	**LytTR**	**PglZ**	**CheY**	**Unclassified**
*F. columnare* ATCC49512	8	9	6	36	9	11	4		1	2	2	3		4	1	1	3
*F. psychrophilum* JIP0286	5	12	9	42	4	11	1			2	4	2	1	5	1	1	4
*F. branchiophilum* FL-15	11	14	8	57	5	11	9	2	1	4	5	2		7	1	2	3
*F. johnsoniae* UW101	41	43	30	171	7	53	14			14	11	8	4	20	1	19	7
*F. indicum* GPTSA100-9	8	20	11	42	5	21	5			4	5	6		9	1	4	8

*OCS, One-component systems; RR, DNA-binding response regulators; SF, sigma factors; TR, transcriptional regulators; ODP; other DNA-binding proteins*.

### Secretion systems

Gram-negative bacteria have several types of secretion systems (Koster et al., 2000; Johnson et al., 2006). *Flavobacterium* secretion systems are shown in Table 3. The T9SS system is unique in functioning as both a secretion system and contributing to gliding motility (McBride and Nakane, 2015). All five of the *Flavobacterium* genomes encode one whole T9SS operon, but the *porQ* gene is present in all *Flavobacterium* genomes twice.

Table 3**Secretion systems distribution in ***Flavobacterium*** genomes**.

**Table:** 

	**T2SS**															**T4P**														**T3SS**											
	**M.G**.									**A.G**.				**F.G**.												**A.G**.	**F.G**.			**M.G**.									**A.G**.		
	**gspD**	**gspE**	**gspF**	**gspG**	**gspM**	**gspH**	**gspI**	**gspJ**	**gspK**	**gspL**	**gspN**	**gspO**	**gspC**	**Tad_tadZ**	**T4P_pilM**	**pilT_piIU**	**piIP**	**piIQ**	**piIAE**	**piIB**	**piIC**	**piII_piIV**	**piIN**	**piIO**	**piIM**	**piID**	**T2SS_gspN**	**Tad_tadZ**	**T2SS_gspC**	**sctC**	**sctJ**	**sctN**	**sctS**	**sctR**	**sctQ**	**sctV**	**sctU**	**sctT**	**FIg_flgC**	**FIg_fliE**	**FIg_flgB**
*Flavobacterium columnare* ATCC 49512								1							1			1		1	1				1							2									
*Flavobacterium psychrophilum* JIP0286	1		1												1				2	1					1							2									
*Flavobacterium branchiophilum* FL-15																									1							2									
*Flavobacterium johnsoniae* UW101																		1		1	1				1							2									
*Flavobacterium indicum* GPTSA100-9																									1							2

**Table:** 

	**T6SSi**														**T6SSii**																	**T6SSiii**											
	**M.G**.														**M.G**.																	**M.G**.											
	**tssA**	**evpJ**	**tssB**	**tssC**	**tssD**	**tssE**	**tssF**	**tssG**	**tssH**	**tssI**	**tssJ**	**tssK**	**tssL**	**tssM**	**pdpB**	**pdpC**	**pdpA**	**pdpD**	**pdpE**	**iglF**	**iglJ**	**iglH**	**iglI**	**vgrG**	**iglB**	**iglC**	**iglA**	**dotU**	**iglG**	**iglD**	**iglE**	**tssD**	**tssE**	**tssF**	**tssG**	**tssB**	**tssC**	**tssN**	**tssH**	**tssI**	**tssK**	**tssP**	**tssQ**
*Flavobacterium columnare* ATCC 49512									2																							8	1	1	1	1	1	1	1	4	1	1	
*Flavobacterium psychrophilum* JIP0286									1																														1				
*Flavobacterium branchiophilum* FL-15									1																							4	1	1	1	1	1	1	2	3	1	1	1
*Flavobacterium johnsoniae* UW101		1							2															1								4	2	1	1	1	1	2	1	5	1	1	
*Flavobacterium indicum* GPTSA100-9									2																														1

**Table:** 

	**T1SS**			**T4SS type B**															**T4SS type T**												**T9SS**										
	**M.G**.			**M.G**.			**A.G**.												**M.G**.			**A.G**.									**M.G**.								**A.G**.		
	**abc**	**mfp**	**omf**	**virb4**	**t4cp1**	**MOBB**	**traP**	**traQ**	**traF**	**traE**	**traN**	**traO**	**traL**	**traM**	**traJ**	**traK**	**traH**	**traI**	**virb4**	**t4cp1**	**MOBB**	**virB11**	**virB6**	**virB5**	**virB10**	**virB3**	**virB2**	**virB1**	**virB9**	**virB8**	**porV**	**sprE**	**sprA**	**gldN**	**gldK**	**sprT**	**gldM**	**gldL**	**gldJ**	**porU**	**porQ**
*Flavobacterium columnare* ATCC 49512	6	3	4																												1	1	1	1	1	1	1	1	1	1	2
*Flavobacterium psychrophilum* JIP0286	7	3	5				1																								1	1	1	1	1	1	1	1	1	1	2
*Flavobacterium branchiophilum* FL-15	5	2	4																								1				1	1	1	1	1	1	1	1	1	1	2
*Flavobacterium johnsoniae* UW101	11	5	15						3	3	3			3	3	3		7									1				1	1	1	2	1	1	1	1	1	1	2
*Flavobacterium indicum* GPTSA100-9	8	5	7																												1	1	1	1	1	1	1	1	1	1	2

*M.G., mandatory genes (genes that must be present in the genome to define this system); A.G., accessory genes (genes that can be present, but do not have to be found in every case); and F.G., forbidden genes (genes that must not be present in the system)*.

The type VI secretion-related pathway (designated subtype III or T6SS^iii^) is restricted to the Bacteroidetes phylum (Russell et al., 2014; Abby et al., 2016), but our comparative analysis showed that *F. psychrophilum* JIP0286 and *F. indicum* GPTSA100-9 do not carry T6SS^iii^. On the other hand, *F. branchiophilum* FL-15 encodes the entire operon of the T6SS^iii^, whereas *F. columnare* ATCC 49512 and *F. johnsoniae* UW101 are missing the *tssQ* gene. Interestingly, one of the components of T6SS^iii^ is TssD, which has eight gene copies in *F. columnare* and four in *F. branchiophilum* and *F. johnsoniae*. TssD is one of the core elements of the T6SS^iii^ injection mechanism, participating in the assembly of the secretion system and export of its effectors (Silverman et al., 2013).

All five of the *Flavobacterium* genomes carry a complete Type I secretion system, which consists of three essential components: ABC-transporter (*abc*), porin (*omf*) and the inner-membrane-anchored adaptor protein (*mfp*). The T1SS is encoded multiple times in the five flavobacterial genomes. T1SS is responsible for secretion of toxins and various other proteins ranging in size from 5.8 to 900 kDa (Kanonenberg et al., 2013).

Interestingly, only the *F. johnsoniae* UW101 genome carries T4SS elements. However, although it carries multiple copies of some T4SS type B accessory elements, *F. johnsoniae* has none of the mandatory T4SS genes, indicating a functional T4SS is not present in any of the *Flavobacterium* genomes. This system is used by some pathogenic bacteria to translocate virulence genes and mediate horizontal gene transfer (Wallden et al., 2010). T3SS, which is important in many Gram-negative pathogens, is not encoded by any of the *Flavobacterium* species analyzed. T2SS is also not encoded by these five species; this system can perform a wide range of functions such as adhesion, signaling, and motility (Nivaskumar and Francetic, 2014). Some of the components of type IV pili (T4P) are encoded by *F. columnare, F. psychrophilum*, and *F. johnsoniae*. However, other critical elements of a T4P system are not encoded by these species. T4P is very similar to T2SS.

### CRISPR-Cas systems analysis

All of the evaluated *Flavobacterium* genomes either carry conserved or possible CRISPR (Clustered regularly interspaced short palindromic repeats)-Cas regions and direct repeat sequences. All the identified CRISPR elements and their components are listed in Table 4. The *Flavobacterium* genomes have CRISPR spacers, and such repeat regions could be the result of the horizontal transfer and contribute to flavobacterial genome plasticity (Lopatina et al., 2016). Some bacteria collected from Antarctic surfaces in a metagenomics study carry more spacers with a high level of diversity, which may result from a need for mobile genetic elements to adapt to extreme Antarctic conditions (Lopatina et al., 2016). CRISPR regions in the *F. columnare* ATCC 49512, *F. psychrophilum* JIP0286, and *F. branchiophilum* FL-15 genomes carry multiple spacer elements ranging in size from 29 to 78 bp.

**Table 4:** **CRISPR-Cas systems analysis of ***Flavobacterium*** genomes**.

***Flavobacterium*** **genomes**	**CRISPR**	**CRISPR candidates**	**Direct repeat at sequence**	**Direct repeat no**	**Spacer size(bp)**	**Start point**	**Length(bp)**	**Cas genes No**.
*Flavobacterium columnare* ATCC 49512	CRISPR-1	Confirmed	GTTGTGGTTTGATTAAAGATTAGAAAACACGATATT	44	29–78	391564	2,912	cas9_TypOeII, cas1_TypeII
	CRISPR-2	Confirmed	GTTGGGAAAGCCCTTATTTTGAAGGGTATCTACAAC	9	30	1679967	563	
	CRISPR-3	Questionable	GTTGTAAATTGCTTTCAATTTTT	2	54	1994455	99	
*Flavobacterium psychrophilum* JIP0286	CRISPR-19	Confirmed	CAATAGAAGTTACAGAATTAGGAAT	5	44	232904	300	cas9_TypeII, cas1_TypeII, cas2_TypeI-II-III
	CRISPR-20	Confirmed	GTTGGTAATTATAAGCTAAAATACAATTTTGAAAGCAATTCACAAC	21	29–30	1325548	1,560	
	CRISPR-21	Questionable	TTAATCTATATTCAAAAATCAAAT	2	30	17425	77	
	CRISPR-22	Questionable	ACCGAACTAAACGCAAAAAAGTGTTGAGA	3	27–56	2020986	170	
	CRISPR-23	Questionable	ACCGAACTAAACGCAAAAAAGTGTTGAGA	3	27–56	2021433	169	
	CRISPR-24	Questionable	TCTCAACACTTTTTTGCGTTCAGTTCGGT	2	56	2026310	113	
	CRISPR-25	Questionable	AGTTTTTTCTCCACACTTTTTTGCGTTCAGTTCGG	2	55	2084863	124	
*Flavobacterium branchiophilum* FL-15	CRISPR-4	Confirmed	CATATCGTGTTTTCTAATCTTTAATCAAACCACAAC	29	29–78	1899989	1,928	
	CRISPR-5	Confirmed	GTTGTAACTGCCCTTATTTTGAAGGGTAAACACAAC	12	29–30	2549336	756	
	CRISPR-6	Confirmed	GTTGTAACTGCCCTTATTTTGAAGGGTAAACACAAC	10	29–30	2550434	628	
	CRISPR-7	Confirmed	GTTGTAACTGCCCTTATTTTGAAGGGTAAACACAAC	6	29–30	2551183	364	
	CRISPR-8	Confirmed	GTTTAAAACCACTTTAAAATTTCTACTATTGTAGAT	38	26–32	3018429	2,392	
	CRISPR-9	Questionable	CTCCTGCAAGGTTTCCAAAACCTTGTAGGTG	3	25-41	85275	158	
	CRISPR-10	Questionable	ATGGAAAAATTATTTGCTTAATCCCCATAAAATTCATGTGAACC	2	31	1392707	118	
	CRISPR-11	Questionable	TTTATTTTTTATAGGTATTCGTT	2	38	1460985	83	
	CRISPR-12	Questionable	ATGGATTTTTATTTTTTATAGGTATTCGTT	2	31	1697623	90	
	CRISPR-13	Questionable	GTTGTAACTGCCCTTATTTTGAAGGGTAAACACAAC	2	29	2550212	101	
	CRISPR-14	Questionable	TAAAAAAAAACAGATAAAAATCCGTGTTTACGAAGTATCCGT	2	58	2858645	141	
	CRISPR-15	Questionable	GGATTTTATTTTTTATAGGTATTCGTT	2	34	3071863	87	
*Flavobacterium johnsoniae* UW101	CRISPR-26	Confirmed	AATCAATTTACAGGTACAATTCC	5	49	644859	310	
	CRISPR-27	Confirmed	ACTTTTACTTAACATCGTTAGATAATTTAAT	4	26–48	5194431	227	
	CRISPR-28	Questionable	AATCAATTTACAGGTACAATTCC	2	49	644499	95	
	CRISPR-29	Questionable	TTAAAACCATATAAATCTATATG	2	36	3182380	81	
	CRISPR-30	Questionable	TTTTATTTCTGAACAAAGATTTCAAAAC	2	61	5739460	116	
*Flavobacterium indicum* GPTSA100-9	CRISPR-16	Questionable	TTCTATGCGCCCCTCTTTGTCAT	2	39	1744952	84	
	CRISPR-17	Questionable	AGAACAAATACTACAACACAACCTAGA	2	51	2058296	104	
	CRISPR-18	Questionable	TACTGTAGGACATTTGTCATCTTTATC	2	60	2872075	113	

Interestingly, only *F. columnare* ATCC 49512 and *F. psychrophilum* JIP0286 genomes carry *cas* genes flanking the CRISPR regions. The *F. columnare* genome carries two different types of *cas* genes; one of them is *cas9*, and the other one is *cas1*. The *F. psychrophilum* genome carries three different types of *cas* genes: *cas1, cas2*, and *cas9*. Bacteria use the CRISPR-Cas system to provide resistance against foreign elements such as plasmids or phages. Cas1 and Cas2 catalyze spacer acquisition from foreign DNA, and Cas9 (also called Csn1) participates in blocking transcription (Nunez et al., 2015).

### Genomic islands

Genomic islands are clusters of genes that often are associated with microbial adaptations of medical or environmental interest (Langille et al., 2008). Often the G+C content of pathogenicity islands differs from that of the rest of the host genome, clearly indicating that those regions originated or adapted from other bacteria by horizontal gene transfer (Dobrindt and Reidl, 2000). Interestingly, G+C content of genomic islands in the flavobacterial genomes did not differ dramatically from the overall genome G+C content (for example, *F. columnare* ATCC 49512 genome G+C content is 31.5% compared to 32.3% for the genomic islands), but genomic islands are still identifiable using other features.

Fourteen genomic islands were identified in *F. columnare* ATCC 49512 ranging from 5 to 30 kb in length. By comparison, *F. johnsoniae* UW101 has 29 predicted genomic islands, *F. psychrophilum* JIP02/86 has five, *F. branchiophilum* FL-15 has seven, and *F. indicum* GPSTA100-9T has eight. All of the genomic islands appear unique; none are shared between species. Genomic islands often carry transmissible genomic elements such as transposons, bacteriophages, or plasmids (Hacker et al., 1997). Three of the *F. columnare* islands include genes encoding integrase, integrase catalytic subunit, or integrase family protein, suggesting horizontal gene transfer was the source of these genomic islands. These genes are commonly found in pathogenicity islands and can be responsible for the formation, integration, deletion, and mobility of pathogenicity islands (Hacker and Kaper, 2000). Putative proteins encoded in the predicted *F. columnare* ATCC 49512 and other *Flavobacterium* genomes' genomic islands are provided in Supplementary Table 3.

### Insertion elements

**Figure 4:**
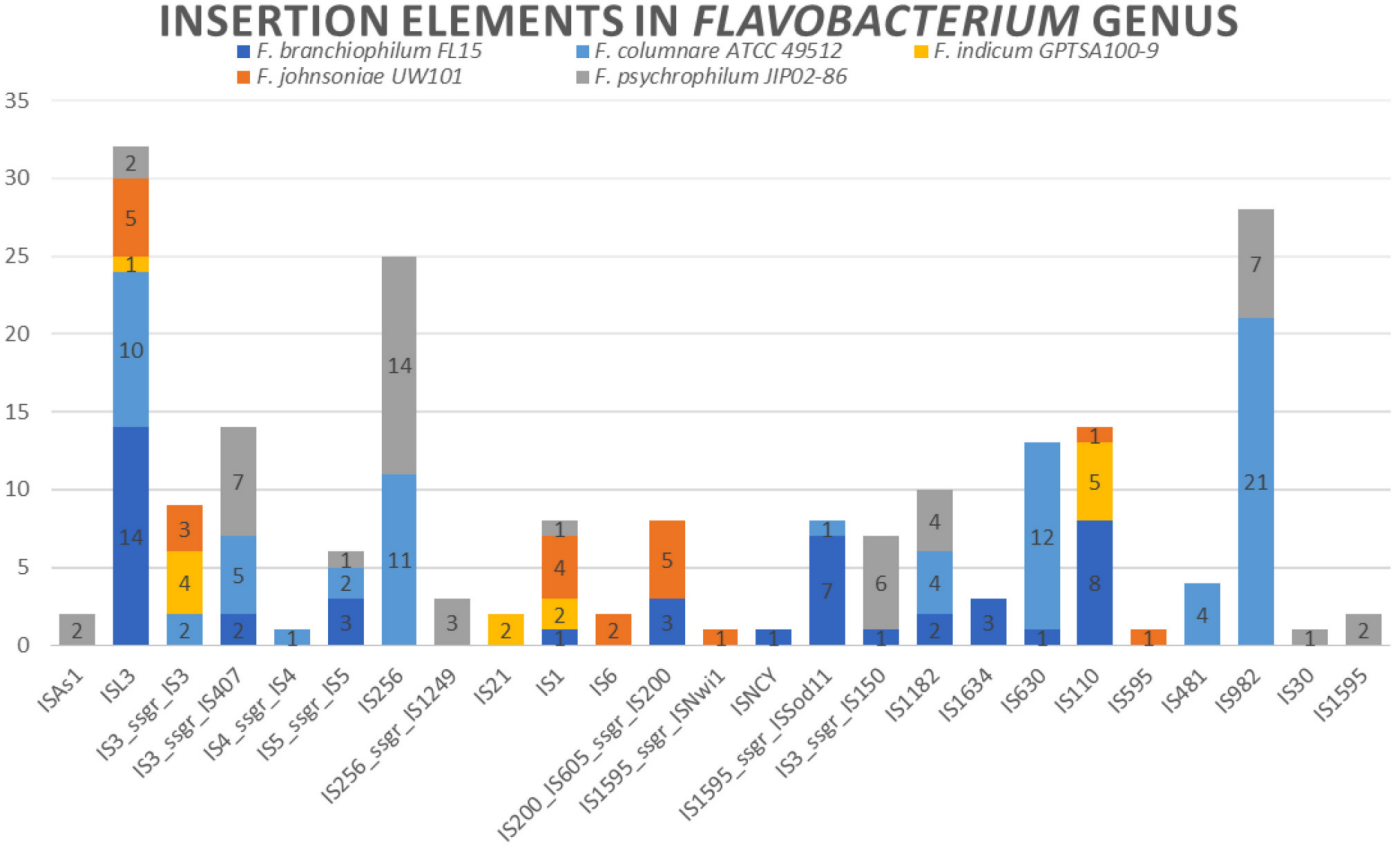
**Insertion elements distribution in ***Flavobacterium*** genomes**.

Insertion elements are widely distributed in bacteria and play an important role in prokaryotic genome evolution and organization due to horizontal gene transfer (Kusumoto et al., 2011). Insertion element expansion can also contribute to genome rearrangement or genome reduction. Eleven different insertion element families in the *F. columnare* ATCC 49512 genome and 73 total insertion elements were identified (Varani et al., 2011). In comparison, *F. psychrophilum* JIP02/86 had 12 families and 50 insertion elements, *F. branchiophilum* FL-15 had 12 families and 46 insertion elements, *F. indicum* GPTSA100-9 had 5 families and 14 insertion elements, and *F. johnsoniae* UW101 had 8 families and 22 insertion elements (Supplementary Table 4) (Figure 4). *F. columnare* ATCC 49512 had the largest number of insertion elements, but *F. branchiophilum* FL-15 and *F. psychrophilum* JIP02/86 had slightly more variety of insertion families. Interestingly, *F. johnsoniae* had fewer insertion elements. Therefore, it appears that the number of flavobacterial insertion elements is not directly related to genome size.

All the *Flavobacterium* genomes carry ISL3 family insertion elements. More than 80 bacterial species carry insertion elements in this family. Genes encoding these insertion elements range in size from 1,186 to 1,553 bp. The typically have imperfect repeats ranging in size from 15 to 39 bp along with 8 bp direct repeats. Additionally, the protein usually includes an α-helical domain (Siguier et al., 2015). In *Pseudomonas stutzeri*, ISL3 elements' translocation can be induced by zygotic induction after conjugative interaction (Christie-Oleza et al., 2010). There is evidence that insertion elements do not always insert randomly but often target preferred sequence elements (Siguier et al., 2014). Target preference for ISL3 elements is not known, but our comparative analysis and general *Flavobacterium* low G+C genome content suggests that ISL3 members could target AT-rich regions.

IS982 and IS256 elements are present only in *F. columnare* ATCC 49512 and *F. psychrophilum* JIP02/86 genomes. In particular, IS256 elements contribute to genome flexibility, genome evolution, and adaptation (Hennig and Ziebuhr, 2010).

### Prophages

Prophages can induce virulence of opportunistic bacteria by activating virulence factors (such as in *Vibrio cholerae*) (Waldor and Mekalanos, 1996). Some bacteria can gain resistance against prophages, which can have the effect of reduced virulence (such as in *Salmonella enterica*) (Rychlik et al., 2001). *F. columnare* ATCC 49512 has one incomplete bacteriophage region (Supplementary Table 5). Three incomplete regions are in the *F. branchiophilum* FL-15 genome, and one incomplete and one questionable region are in the *F. johnsoniae* UW101 genome. Only the *F. psychrophilum* JIP02/86 genome carries a complete phage region, which has been previously described (Castillo et al., 2014) and is absent in other *Flavobacterium* genomes (Wiens et al., 2014). *F. indicum* GPTSA100-9 genome does not carry any predicted bacteriophage regions.

The incomplete *F. columnare* phage region size is ~19 kb, and it contains 18 ORFs. Most encode hypothetical proteins, but some of the ORFs encode proteins with predicted functions, including AttL, phage tail tape measure protein, and phage lysin proteins. The incomplete phage regions in *F. branchiophilum* FL-15 are 10.5 kb (12 ORFs), 6.8 kb (7 ORFs), and 7.5 kb (8 ORFs), and they include ORFs encoding chemical-damaging agent resistance protein and toxic ion resistance protein, which are unique to this genome. The incomplete phage region in *F. johnsoniae* UW101 has 21 ORFs (15.6 kb), and one of its ORFs encodes arsenate reductase. *F. columnare, F. psychrophilum*, and *F. johnsoniae* phage regions all include *attL* gene, and *F. psychrophilum* and *F. johnsoniae* also carry the *attR* gene. AttL and AttR proteins are responsible for creating recombinant junctions with the help of phage integrase gene.

The phage region in *F. psychrophilum* JIP02/86 is classified as bacteriophage type 6H, which is an abundant type of temperate phage that is lysogenized in a large number of *F. psychrophilum* strains (Castillo et al., 2014). It is a 47-kb phage region that has 64 ORFs (Supplementary Table 5). One of the ORFs encodes LuxR family protein, which is present only in the *F. psychrophilum* genome. The majority of the ORFs in the *F. psychrophilum* phage region encode hypothetical proteins.

### Predicted virulence factors

The *Flavobacterium* genomes are valuable tools for identification of potential virulence mechanisms. To be considered candidate virulence factors in the current study, *Flavobacterium* proteins had to have BLAST result in MvirDB with an *E*-value <1^*^10^−10^. Using these criteria, *F. columnare* ATCC 49512 encodes 567 potential virulence proteins, *F. branchiophilum* FL-15 has 604, *F. indicum* GPTSA100-9 encodes 560, *F. johnsoniae* UW101 has 1141, and *F. psychrophilum* JIP02/86 has 557 (Supplementary Table 6). However, it should be noted that sequence similarities to proteins in MvirDB, which is a repository of microbial virulence proteins, does not necessarily indicate that the identified flavobacterial proteins have the same virulence functions. In particular, *F. indicum* and *F. johnsoniae* are not known to be pathogenic, so it is likely that many of these protein matches from these species do not function in virulence.

An example of a putative virulence factor in this list is KatG (catalase-peroxidase), which is present in *F. columnare* ATCC 49512 and other *Flavobacterium* genomes. KatG can enable resisting the oxidative burst in phagocytes. In the fish pathogen *Edwardsiella tarda*, KatG provides protection against endogenous and exogenous H_2_O_2_,and it is involved in pathogenesis (Xiao et al., 2012). Another example is GdhA (glutamate dehydrogenase), which is encoded in the *F. columnare* ATCC 49512 genome and other *Flavobacterium* genomes. GdhA is important in bacterial nitrogen metabolism; in *Streptococcus pneumoniae*, GdhA is expressed in nitrogen limitation and is tightly regulated by virulence proteins GlnR and CodY (Hendriksen et al., 2008). Another potential virulence gene identified encodes ClpP protease, which plays an important role in stress response and biofilm formation in pathogenic bacteria. Mutation of this gene in *Actinobacillus pleuropneumoniae* showed that it mediates tolerance to several stressors and regulates virulence and iron utilization (Xie et al., 2013).

Several proteins with matches in MvirDB are encoded in all the evaluated *Flavobacterium* genomes. These include alginate o-acetyltransferase (AlgI), ABC transporter ATP-binding proteins, aspartate alpha-decarboxylase, bacteriophage components, capsular polysaccharide synthesis enzymes, cold shock-like proteins, CTP synthetize, ferric uptake regulators, glutamate dehydrogenase, invasion regulatory proteins, and thioredoxin reductase (TrxB).

*F. columnare* ATCC 49512 encodes 89 unique potential virulence proteins that are not in the other four flavobacterial genomes. Among these are a catalase and peroxiredoxin, which are both involved in bacterial resistance to toxic peroxides. A TonB-dependent siderophore receptor is also unique to *F. columnare*, as well as a thiol-activated cytolysin that is similar to listeriolysin O and streptolysin O. Several polysaccharide envelope synthesis enzymes are unique to *F. columnare*, including an N-acetyl neuramic acid synthase, a UDP-N-acetylglucosamine-2-epimerase, glycosyl transferases, and capsular polysaccharide synthesis enzymes. Several transcriptional regulators and sensor histidine kinases are also unique to *F. columnare* ATCC 49512.

### Metabolism

Metabolic pathways of *F. psychrophilum* have been extensively described (Duchaud et al., 2007). Pathway analyses using the Kyoto Encyclopedia of Genes and Genomes (KEGG) Pathway Database (Ogata et al., 1999; Kanehisa and Goto, 2000) revealed that *F. columnare* ATCC 49512 genome has a complete tricarboxylic acid (TCA) cycle and glycolysis pathway. All of the other *Flavobacterium* strains in our comparative analysis also have complete TCA and glycolysis pathways. However, some carbohydrate metabolism components are unique to *F. johnsoniae* such as pentose phosphate pathway and pentose/glucuronate interconversion elements. Moreover, pathway comparison showed that starch and sucrose utilization pathways are present only in *F. johnsoniae* and *F. branchiophilum*.

*F. columnare* ATCC 49512 genome has predicted protein functions in oxidative phosphorylation, which is driven by the electron transport chain and TCA cycle (McNeil and Fineran, 2013). Oxidative phosphorylation consists of five different complexes, and our comparative analysis showed that all *Flavobacterium* strains encode oxidative phosphorylation components except for two missing genes. One of the missing genes encodes a protein in complex II, which is an important respiratory enzyme participating in both electron transport chain and TCA cycle. Complex II consists of four subunits: SdhA, SdhB, SdhC, and SdhD. SdhD is a hydrophobic membrane anchor and is missing in all the *Flavobacterium* strains (McNeil and Fineran, 2013). *F. johnsoniae* is also missing one gene encoding a protein from complex IV, cytochrome c oxidase cbb3-type subunit III. *F. johnsoniae* and *F. branchiophilum* also encode two cytochrome bd complex proteins (CydA and CydB) that are missing in the other *Flavobacterium* species.

*F. columnare* and *F. johnsoniae* are unique among the sequenced *Flavobacterium* species in that they encode more proteins in nitrogen metabolism. The other sequenced *Flavobacterium* strains do not carry 80% of the nitrogen metabolism genes carried by *F. columnare* and *F. johnsoniae*. In particular, *F. columnare* ATCC 49512 encodes protein functions suggesting it is capable of denitrification, which results in a reduction of nitrate and nitrite to molecular nitrogen (N_2_). It has predicted protein functions indicating that it is capable of nitrous oxide reduction, nitric oxide reduction, nitrite reduction, nitrate reduction, and nitrite/nitrate transport. Nitrate and nitrite are abundant in eutrophic freshwater environments where *F. columnare* is commonly found, particularly in warm water aquaculture systems where it is a common fish pathogen. Its ability to utilize nitrate and nitrite as electron acceptors for the electron transport system suggest that it is capable of actively metabolizing and multiplying in pond sediments that are commonly oxygen depleted or anaerobic.

Interestingly, *F. columnare* has a complete urea cycle, but it does not encode a urease. Ammonia is typically abundant in eutrophic freshwater environments, but the advantage to *F. columnare* of converting ammonia to urea is not currently clear. In *Helicobacter pylori*, it appears the urea cycle is used to maintain intrabacterial nitrogen balance (Mendz and Hazell, 1996). In marine diatoms growing in nutrient-rich environments, the urea cycle is important for the response of diatoms to episodic changes in nitrogen availability (Allen et al., 2011).

Fatty acids are crucial components of membranes and play an important role in energy metabolism. To maintain membrane lipid homeostasis, fatty acid biosynthesis and degradation pathways must be controlled coordinately (Fujita et al., 2007). Our metabolic pathway analysis showed that all the sequenced *Flavobacterium* species have fatty acid biosynthesis, whereas fatty acid degradation pathway is present only in the *F. johnsoniae* genome.

Bacteria require iron as an essential nutrient. Iron acquisition is particularly important for pathogenic bacteria because of limited availability inside the host (Beaz-Hidalgo and Figueras, 2013). Therefore, the iron acquisition is linked to bacterial virulence (Guan et al., 2013). Gram-negative bacteria iron uptake mechanisms have been reviewed (Brown and Holden, 2002). Recently, two iron acquisition elements were described from *F. columnare* (Guan et al., 2013). One of them was TonB-dependent ferrichrome-iron receptor precursor FhuA, and the second was putative ferric uptake regulator Fur. We identified a predicted gene encoding FhuA in *F. columnare* ATCC 49512, and the genome also has three iron ABC transporter genes encoding FhuD, FhuB, and FhuC. Also, a gene encoding ferric iron uptake transcriptional regulator (Fur) is encoded in the *F. columnare* genome. *F. johnsoniae, F. psychrophilum, F. branchiophilum*, and *F. indicum* have the same iron acquisition elements.

### Gliding motility

Bacterial gliding motility is an energy requiring process of bacterial translocation over a surface. In this process, flagella are not required, and bacterial movement is along the long axis of the bacterium. Gliding motility results in thin spreading edges on colonies (McBride, 2001), and it is considered one of the characteristics of some species in the phylum Bacteroidetes. Many of the motility genes from this phylum are novel and are not found outside the Bacteroidetes. There are two major protein systems responsible for gliding motility. First, Gld proteins are components of the “motor” that moves the cell. Proteins encoded by *gldA, gldB, gldD, gldF, gldG, gldH, gldI, gldJ, gldK, gldL, gldM*, and *gldN* are required for gliding (Nelson et al., 2008). Second, Spr proteins are cell surface proteins responsible for adhesion or protein secretion (McBride et al., 2009). Deletion or disruption of genes encoding Gld proteins results in loss of motility, but *spr* deletion causes partial loss of gliding motility (Nelson et al., 2008).

Gliding motility of flavobacteria is well described (Bernardet et al., 1996; Decostere et al., 1998), particularly in *F. johnsoniae*. However, it has not been characterized in *F. columnare*. In the *F. columnare* ATCC 49512 genome, 13 gliding motility genes were identified (including a gene encoding GldL, which is currently annotated as a hypothetical protein). GldE gene is missing in *F. columnare* ATCC 49512, *F. psychrophilum* JIB02/86, and *F. indicum* GPSTA100-9T. In *F. johnsoniae*, overexpression of *gldE* gene partly suppressed the motility defects of a *gldB* mutant, suggesting that *gldB* and *gldE* might have overlapping functions (Hunnicutt and McBride, 2001). Interestingly, GldO is present only in *F. johnsoniae* UW101; this protein is similar to GldN, and the two proteins can partially replace each other for function (Rhodes et al., 2010). Genes encoding SprA, SprB, SprC, SprD, SprE, SprF, and SprT were identified in all the evaluated *Flavobacterium* genomes. Moreover, like most *Flavobacterium* species, they encode multiple paralogs of some *spr* genes in their genomes.

### *F. columnare* anaerobic growth

Denitrification occurs in the absence of O_2._Nitrate serves as an electron acceptor for anaerobic respiration. Although not as efficient as aerobic respiration, it provides microbes with a relatively high amount of energy. In bacteria with denitrification capability, nitrate reduction is mediated by one of two enzymes; one is a membrane-bound nitrate reductase, and the other is a soluble cytoplasmic nitrate reductase (Gennis and Stewart, 1996). The latter is encoded in the genome of *F. columnare* ATCC 49512. Subsequently, the reduction of nitrite to dinitrogen through nitric oxide and nitrous oxide is catalyzed by reductases encoded by *nirK, norB*, and *nosZ*.

**Figure 5:**
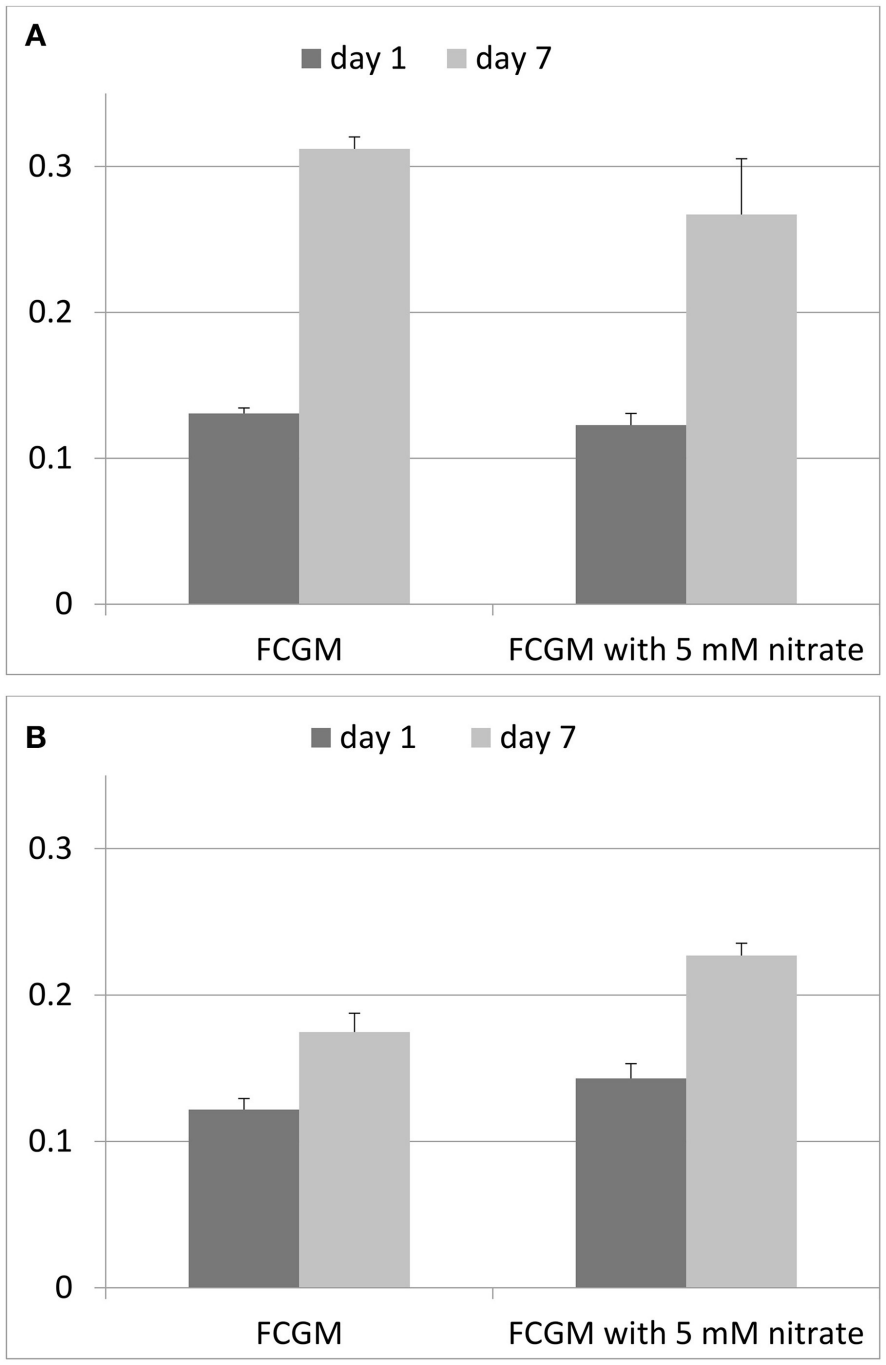
**Growth of ***F. columnare*** ATCC 49512 in FCGM and FCGM supplemented with 5 mM nitrate. (A)** Growth under aerobic conditions and **(B)** growth under anaerobic conditions.

*F. columnare* ATCC 49512 has the genetic capability to completely reduce nitrate into nitrogen due to the presence of genes encoding all the denitrification reductases (Tekedar et al., 2012), suggesting that *F. columnare* ATCC 49512 can grow anaerobically using nitrogenous compounds as electron acceptors for anaerobic respiration. In the present study, we compared *F. columnare* ATCC 49512 growth under aerobic and anaerobic conditions in presence and absence of nitrate supplementation (Figure 5). Our results indicate that nitrate did not improve aerobic growth, but it did improve anaerobic growth. As expected, anaerobic growth with nitrate supplementation was not as efficient as aerobic growth. However, our results suggest for the first time that *F. columnare* is capable of anaerobic growth, and that anaerobic growth is improved in the presence of 5 mM sodium nitrate. Extracellular nitrite production was not detected, indicating this intermediate product was likely subsequently reduced to nitric oxide without accumulation.

### Transcriptome analysis

A total of 63,390,073 reads were aligned to the genome using Bowtie 2 with an average 98% alignment per sample. Genome annotation for *F. columnare* was downloaded from NCBI FTP and used for analysis of expression with BIRAP. From the expression profile generated by RNA-seq, a basal expression cutoff was calculated per million reads for each sample. Of the 2,772 annotated genes, BIRAP identified 2,635 as expressed above baseline. Of the 137 that were not expressed, 64 were annotated as hypothetical proteins (~47%).

A total of 1,132 EIRs of size 70 bp or more were identified by BIRAP and were analyzed. BlastX search against the non-redundant bacterial database identified 41 that had matches with annotated bacterial proteins in other species (Table 5), 16 of which had a non-traditional start site (TTG, GTG, and CTT). Thirty-six of these proteins matched with conserved hypothetical proteins, while a few proteins matched with proteins that had annotated functions defined. Identified protein functions include an ATPase, a DUF4295 domain-containing protein, molybdopterin molybdenum transferase MoeA, rod shape-determining protein MreD, and a Teichoic-acid-transporting ATPase. All 41 proteins were searched against virulence database MvirDB (Zhou et al., 2007), and 30 of them had matches, suggesting potential roles in virulence (Table 5).

**Table 5:** **Novel protein coding regions**.

**Start**	**Stop**	**Strand**	**Length (aa)**	**Blast hit**	**Description**	**Start codon**	**MVIRDB/Organism**
39037	39636	−	200	WP_065213772.1	Hypothetical protein		No hit
85376	85573	−	66	ANO48202.1	Hypothetical protein Pf1_02748	TTG	No hit
95864	96004	+	47	ANO48210.1	Hypothetical protein Pf1_02756		CNF modifies all members of the Rho family of small GTPases, leading to their activation
134375	124572	+	66	WP_065213298.1	Hypothetical protein	CTT	capsular polysaccharide biosynthesis protein Cps4D [Streptococcus pneumoniae TIGR4]
141945	142067	+	41	ANO48250.1	Hypothetical protein Pf1_02796	TTG	No hit
200731	200895	+	55	ANO49209.1	Hypothetical protein Pf1_00961		hypothetical protein Z5095 [Escherichia coli O157:H7 EDL933]
468693	469853	+	387	WP_014164538.1	Molybdopterin molybdenumtransferase MoeA		Vacuolating cytotoxin;
554290	554439	−	50	ANO48894.1	Hypothetical protein Pf1_00646	GTG	toxin co-regulated pilus biosynthesis protein T [Vibrio cholerae O1 biovar El Tor str. N16961]
594135	594797	−	221	WP_065213557.1	ATPase		AatC ATB binding protein of ABC transporter [Escherichia coli]
680568	680756	−	63	ANO49370.1	Hypothetical protein Pf1_01125	TTG	Zinc metalloproteinase/disintegrin
692884	693144	−	87	ANO49363.1	Hypothetical protein Pf1_01118		trans-sialidase [Trypanosoma cruzi strain CL Brener]
727112	727264	−	51	ANO49336.1	Hypothetical protein Pf1_01091		Group III snake venom metalloproteinase
744844	744960	+	39	ANO49317.1	Hypothetical protein Pf1_01072		two-component system sensor histidine kinase [Bacteroides thetaiotaomicron VPI-5482]
745653	745790	+	46	ANO49315.1	Hypothetical protein Pf1_01070	TTG	Streptokinase
973850	974353	+	168	WP_065212991.1	Rod shape-determining protein MreD		No hit
1067797	1067946	−	50	ANO47321.1	Hypothetical protein Pf1_01864		No hit
1171749	1171907	+	53	ANO47901.1	Hypothetical protein Pf1_02447	TTG	hypothetical protein XF1753 [Xylella fastidiosa 9a5c]
1404311	1404457	+	49	ANO48160.1	Hypothetical protein Pf1_02706		chloramphenicol acetyltransferase [Vibrio cholerae 1587]
1583470	1583604	−	45	ANO48610.1	Hypothetical protein Pf1_00362	TTG	erythrocyte membrane protein 1 PfEMP1 [Plasmodium falciparum 3D7]
1583723	1583881	−	53	ANO48609.1	Hypothetical protein Pf1_00361	TTG	No hit
1606964	1607101	−	46	ANO48589.1	Hypothetical protein Pf1_00341		No hit
1682815	1682925	+	37	ANO48526.1	Hypothetical protein Pf1_00278	TTG	No hit
1691105	1691263	−	54	ANO48516.1	Hypothetical protein Pf1_00268		Outer membrane protein
1712008	1712136	+	43	ANO48500.1	Hypothetical protein Pf1_00252	TTG	No hit
1848535	1848669	+	45	ANO49588.1	Hypothetical protein Pf1_01346	TTG	flagellum-specific ATP synthase [Campylobacter jejuni subsp. jejuni NCTC 11168]
1973301	1973432	−	44	ANO49696.1	Hypothetical protein Pf1_01455	TTG	minor fimbrial subunit [Salmonella typhimurium LT2]
1975257	1975415	−	53	ANO49696.1	Hypothetical protein Pf1_01458	TTG	minor fimbrial subunit [Salmonella typhimurium LT2]
1994443	1994565	−	41	ANO49717.1	Hypothetical protein Pf1_01476		Phosphoenolpyruvate-Protein Phosphotransferase Phosphotransferase System,Enyzme I; EC = 2.7.3.9
2131190	2131318	−	43	ANO47037.1	Hypothetical protein Pf1_01580		SecD/SecF fusion protein
2142368	2142484	−	39	ANO47045.1	Hypothetical protein Pf1_01588	TTG	Hemolysin-activating lysine-acyltransferase
2172014	2172355	−	114	WP_065212880.1	Hypothetical protein		erythrocyte membrane protein 1 PfEMP1 [Plasmodium falciparum 3D7]
2174006	2174338	−	111	WP_036217889.1	Hypothetical protein		FUR protein
2216770	2216907	+	46	ANO47112.1	Hypothetical protein Pf1_01655		No hit
2298050	2298268	+	73	ANO47180.1	Hypothetical protein Pf1_01723		flavin-binding monooxygenase-like, L-ornithine 5-monooxygense
2472639	2472761	−	41	ANO47787.1	Hypothetical protein Pf1_02332		Arginine succinyltransferase
2684501	2684620	+	40	ANO47598.1	Hypothetical protein Pf1_02143		No hit
2775026	2775151	+	42	ANO47538.1	Hypothetical protein Pf1_02083		putative ABC exporter outer membrane component [Salmonella typhimurium LT2]
2847516	2847671	−	52	ANO48075.1	Hypothetical protein Pf1_02621		putative O-antigen acetylase
2848787	2848936	−	50	WP_014166478.1	DUF4295 domain-containing protein		merozoite surface antigen 2c [Babesia bovis]
2898382	2898615	+	78	WP_014166523.1	Hypothetical protein	TTG	motor torque providing
2947619	2948902	+	428	ANO48353.1	Teichoic-acid-transporting ATPase		ABC transporter, ATP-binding protein [Enterococcus faecalis V583]

Some of the EIRs were adjacent to the 5′ end of annotated genes in the *F. columnare* ATCC 49512 genome, suggesting they could represent start codon corrections. BlastX searches of these EIRs resulted in updated start sites of 25 annotated protein coding regions (Supplementary Table 7), three of which (WP_041253481.1, WP_014166307.1, WP_041253585.1) used an alternate start codon (TTG). Of the 25 protein coding regions, 15 were hypothetical, and 10 had annotated functions.

EIRs with promoters or terminators associated with their loci and that did not have protein matches by BlastX were considered candidate putative non-coding RNAs (Supplementary Table 8). These were searched against Rfam database, but none of the putative non-coding RNAs matched conserved non-coding RNAs in Rfam.

Operons in the *F. columnare* ATCC 49512 genome were predicted using DOOR2. Our RNA-seq data enables experimental evaluation of these predictions. A total of 546 operons with two or more proteins were predicted by DOOR2, and RNA-seq confirmed expression of 526 operons. The complete operon structure determined by RNA-seq and comparison against DOOR2 predictions is presented in Supplementary Table 9.

## Discussion

In this study, we aimed to compare the complete genome sequence of *F. columnare* ATCC 49512 against four other closed and annotated representative flavobacterial genomes to identify unique features and increase our understanding of this important fish pathogen. One of the novel capabilities we identified in the *F. columnare* ATCC 49512 genome is anaerobic respiration using nitrate, nitrite, nitric oxide, and nitrous oxide as electron acceptors. We confirmed this capability experimentally. We also aimed to confirm the predicted annotation using RNA-seq and identify expressed intergenic regions not predicted by our automated annotation method. Our results revealed that RNA-seq is an important tool to improve genome annotation by identification of unannotated intergenic expressed regions, including sRNAs.

Bacterial pan-core and singletons analysis have been used extensively to understand genomic variation between species. The *F. johnsoniae* genome had the greatest contribution of singletons in our analysis, increasing pan-genome size from 5,506 to 8,344 genes, which reflects its unique metabolism adapted to environmental growth. The pan-genome to core genome ratio of the flavobacterial genomes is relatively high compared to other pan-genome analyses such as *Propionibacterium acnes* (Tomida et al., 2013) and *Erwinia amylovora* (Mann et al., 2013). This likely reflects that our analysis was at the genus level, and it also reflects the variety of environmental niches for the flavobacterial species we analyzed. However, despite the variety of their lifestyles, the flavobacterial genomes also had a significant group of core functions (1,252 genes), reflecting similar adaptations to aquatic environments and conserved metabolic strategies for survival. The large pan-genome size of the *Flavobacterium* species may also reflect significant horizontal gene acquisition; in the aquatic and fish host environments, flavobacteria are expected to be exposed to a variety of bacterial species.

Analysis of the *F. columnare* ATCC 49512 genome revealed it has similar core metabolic functions as other flavobacterial genomes, including a complete TCA cycle and glycolysis pathway. Fatty acid biosynthesis pathways are encoded in the *F. columnare* ATCCC 49512 genome, whereas the fatty acid degradation pathway is missing. This indicates that *F. columnare* relies more on carbohydrates and proteins for energy than lipids. Like the other flavobacteria, *F. columnare* ATCC 49512 encodes oxidative phosphorylation functions. However, unlike the other flavobacteria, *F. columnare* is unique in having the capability of using alternate electron acceptors for respiration. Namely, it encodes proteins whose functions indicate *F. columnare* is capable of denitrification, which would allow it to utilize nitrate, nitrite, nitric oxide, and nitrous oxide as electron acceptors for the electron transport system. This suggests that *F. columnare* is capable of anaerobic growth in aquatic pond sediments, which are typically nitrogen rich. This provides important new information on the potential environmental reservoir for *F. columnare* in aquaculture ponds. Our study is the first to identify this predicted capability, and we have now confirmed experimentally that *F. columnare* can grow anaerobically. Furthermore, anaerobic growth is more efficient in the presence of nitrate supplementation, indicating that *F. columnare* is using nitrate as an electron acceptor for anaerobic respiration.

The *F. columnare* ATCC 49512 genome revealed that the species has several other unique metabolic capabilities. Besides denitrification, the *F. columnare* ATCC 49512 genome uniquely encodes nickel permease function, which could play an important role in several cellular processes such as energy metabolism, nitrogen metabolism, detoxification, and virulence (Schauer et al., 2007). *F. columnare* ATCC 49512 is also unique in encoding neprilysin, which is an endopeptidase considered to have a nutritional role in bacteria. Also, it is a membrane-bound protein that is responsible for degradation of biologically active peptides (Rawlings and Salvesen, 2013).

*F. columnare* ATCC 49512 has similar iron acquisition strategies as the other *Flavobacterium* species, and it also has a similar gliding motility system encoded in its genome. However, the flavobacteria vary in the *spr* genes they encode, which reflects variety in the types of surfaces they are required to use as substrates for motility (McBride et al., 2009).

Fish pathogenic strains *F. branchiophilum* FL-15, *F. columnare* ATCC49512, and *F. psychrophilum* JIP0286 carry fewer transcriptional factors and two-component systems elements than environmental isolates *F. indicum* GPTSA100-9 and *F. johnsoniae* UW10. Maintaining a large regulatory network has high genetic cost (Capra and Laub, 2012), and the number of regulatory systems correlates with the number of environmental niches bacteria are required to survive in. The *F. johnsoniae* UW101 transcriptional network is particularly noteworthy for being large relative to the size of its genome (Visweswariah and Busby, 2015), which likely reflects the need to adapt to a variety of environmental conditions. By contrast, the host environment is relatively constant compared to the aquatic environment, so *F. columnare, F. psychrophilum*, and *F. branchiophilum* appear to require fewer regulatory systems. However, the host environment is stressful and requires rapid adaptation to varying host defense systems, so the regulatory functions they carry are critical for survival. For example, *F. psychrophilum* senses temperature with a two-component system that activates virulence-related genes at its optimal growth temperature (Hesami et al., 2011).

All the evaluated *Flavobacterium* genomes carry T9SS and T1SS. The T9SS is important for gliding motility, which is a common feature of flavobacteria. Notably, the flavobacteria do not have a T2SS, which is the main terminal branch of the general secretory system. However, only some of the *Flavobacterium* species carry T6SS^iii^. *F. branchiophilum* FL-15 encodes an entire T6SS, and *F. johnsoniae* UW101 and *F. columnare* ATCC 49512 carry almost the entire T6SS. *F. psychrophilum* JIP0286 and *F. indicum* GPTSA100-9 genomes do not carry T6SS^iii^. The T6SS is particularly known for delivering anti-bacterial toxins, giving a competitive advantage in multi-species environments.

CRISPR-Cas systems are small RNA-based systems that protect bacteria from foreign DNA (bacteriophage and plasmids). A CRISPR segment consists of three elements: CRISPR array, flanking regions consisting of short direct repeats separated by short variable DNA sequences and cas genes, and short repeat regions (Makarova et al., 2015). CRISPR arrays store “memory” sequences using spacers (short DNA sequences), which originate from foreign DNA (Amitai and Sorek, 2016). Because of their unique ecological environments, it is expected that each species will carry different memory sequences depending on the types of foreign DNA they are exposed to. However, only the two pathogens *F. columnare* and *F. psychrophilum* carry genes encoding Cas enzymes that catalyze acquisition of spacer sequences from foreign DNA, possibly indicating more pressure from foreign DNA. The mechanism used by *F. indicum* GPTSA100-9, *F. branchiophilum* FL-15, and *F. johnsoniae* UW101 to obtain CRISPR regions and direct repeat elements is not known because they do not carry *cas* genes.

Despite carrying *cas* genes, *F. columnare* ATCC 49512 had the largest number of insertion elements and second highest number of genomic islands, indicating a relatively high degree of horizontal gene exchange and plasticity among the flavobacterial genomes. As expected, *F. johnsoniae*, the species with the largest genome, had the largest number of genomic islands, but it had surprisingly few insertion elements. *F. branchiophilum* FL-15 and *F. psychrophilum* JIP02/86 had fewer insertion elements than *F. columnare* ATCC 49512, but had a similar variety of insertion families. ISL3 elements are the most distributed family of insertion sequences in the *Flavobacterium* genomes we evaluated. Prophages are not as prevalent in flavobacterial genomes as other bacterial species, with only one (*F. psychrophilum* JIP02/86) carrying a complete phage region. The other four species only carry remnants or incomplete phage regions, with three incomplete regions in *F. branchiophilum* FL-15.

Few *F. columnare* virulence factors have been identified. An initial characterization of *F. columnare* iron acquisition proteins was described (Guan et al., 2013). Biofilm formation in the three *F. columnare* genomovars was compared (Cai et al., 2013). In addition, development of rifampicin-resistant mutants from a virulent *F. columnare* genomovar II strain caused loss of virulence, but the mechanism for attenuation was not identified (Mohammed et al., 2013). Genomic analysis revealed *F. columnare* potential virulence genes and possible virulence mechanisms that warrant further investigation. Some of these potential virulence genes are common to the *Flavobacterium* species, and many are unique to *F. columnare* ATCC 49512. In particular, peroxide resistance, iron metabolism, and stress response were revealed as potentially important virulence mechanisms. Exotoxins/cytotoxins were also identified that could contribute to virulence, and the role of polysaccharide coat in columnaris disease is not known.

Finally, we used RNA-Seq to improve the *F. columnare* ATCC 49512 genome annotation. Using this method, we identified 41 unannotated protein-coding genes, 16 of which had a non-traditional start site (TTG, GTG, and CTT). Also, RNA-seq provided experimental validation for predicted protein-coding ORFs that were identified using automated annotation for the *F. columnare* ATCC 49512 genome (particularly hypothetical proteins), and it allowed analysis of operon structure within the genome. RNA-seq also enables identification of small non-coding RNAs, which are not annotated using automated pipelines. We identified candidate sRNAs in the *F. columnare* ATCC 49512 genome, none of which had matches in the Rfam database. This is likely due to a paucity of data on sRNAs in the Bacteroides group, and we consider this to be the first experimental identification of sRNAs in the *Flavobacterium* genus. Based on our results, we advocate RNA-seq as a relatively low-cost method to improve annotation of reference genomes, particularly in identifying novel proteins and sRNAs that are not identified by automated annotation pipelines.

In summary, the *F. columnare* ATCC 49512 genome revealed several conserved functions the species shares with the other *Flavobacterium* species. Like the other flavobacteria, *F. columnare* likely derives energy primarily from carbohydrates and proteins rather than lipids, and it has similar gliding motility functions. However, unlike the other flavobacteria, *F. columnare* has the genetic capability of utilizing nitrate and other nitrogen molecules for anaerobic respiration. We present experimental evidence to phenotypically substantiate this capability. *F. columnare* ATCC 49512 also has evidence for more horizontal gene exchange than the other flavobacterial species, as evidenced by a high number of insertion sequences and genomic islands. RNA-seq improved the *F. columnare* ATCC 49512 annotation and added value by validating automated annotation and adding operon structure. We also report for the first-time candidate non-coding small RNAs in *F. columnare*.

## Author contributions

HT, AK, JR, and ML conceived and designed the experiments. HT, JR, SK, and SN performed the experiments and analyzed the data. HT wrote and ML and AK edited the manuscript. All authors approve submission of this manuscript to Frontiers in Microbiology.

### Conflict of interest statement

The authors declare that the research was conducted in the absence of any commercial or financial relationships that could be construed as a potential conflict of interest.

## Supplementary material

Supplementary Table 1 **Unique and shared genes for all the ***Flavobacterium*** species**.

Supplementary Table 2 *****Flavobacterium*** transcriptional factors and two-component systems**.

Supplementary Table 3 **Genomic islands in ***Flavobacterium*** genomes**.

Supplementary Table 4 *****Flavobacterium*** genus insertion elements**.

Supplementary Table 5 **Prophage elements in ***Flavobacterium*** genus**.

Supplementary Table 6 **Potential virulence factors in ***Flavobacterium*** genomes**.

Supplementary Table 7 *****F. columnare*** ATCC 49512 proteins with updated start sites based on transcriptome analysis**.

Supplementary Table 8 *****F. columnare*** ATCC 49512 putative non-coding RNAs based on transcriptome analysis**.

Supplementary Table 9 *****F. columnare*** ATCC 49512 operons predicted by DOOR2 and evidence of expression**.
