# Supplementary material for: Comparative Genomics and Transcriptional Analysis of Flavobacterium columnare Strain ATCC 49512
Source: Front Microbiol. 2017 Apr 19;8:588. doi: 10.3389/fmicb.2017.00588 (PMC5395568; doi:10.3389/fmicb.2017.00588)
Supplement: Supplementary Table 7 — F. columnare ATCC 49512 proteins with updated start sites based on transcriptome analysis. [file Table7.DOCX]

**Supplementary Table 7. *F. columnare* ATCC 49512 proteins with updated start sites based on transcriptome analysis.**

| **PROTEIN** | **Start** | **Stop** | **Strand** | **NEW_START** | **NEW_STOP** | **LENGTH (AA)** | **NEW_LENGTH (AA)** | **BLAST HIT** |
| --- | --- | --- | --- | --- | --- | --- | --- | --- |
| WP_041253304.1 | 145141 | 146094 | - | 145141 | 146133 | 317 | 330 | ANO48252.1 |
| WP_014164353.1 | 270195 | 270950 | + | 270138 | 270950 | 251 | 270 | ANO49152.1 |
| WP_014164379.1 | 298785 | 299138 | - | 298785 | 299195 | 117 | 136 | WP_041253150.1 |
| WP_041253321.1 | 354613 | 355683 | - | 354613 | 355815 | 356 | 400 | ANO49080.1 |
| WP_050855764.1 | 428994 | 430092 | - | 428994 | 430179 | 382 | 411 | WP_014164497.1 |
| WP_014164580.1 | 432376 | 433197 | + | 432196 | 433197 | 273 | 344 | ANO49003.1 |
| WP_014164532.1 | 459681 | 460112 | - | 459681 | 460373 | 143 | 230 | ANO48954.1 |
| WP_041253345.1 | 707359 | 708465 | - | 707359 | 708660 | 368 | 433 | ANO49353.1 |
| WP_014165046.1 | 1113503 | 1119325 | + | 1113386 | 1119325 | 1940 | 1979 | WP_063743397.1 |
| WP_050855818.1 | 1122412 | 1122843 | + | 1121527 | 1122843 | 143 | 438 | ANO47849.1 |
| WP_041253392.1 | 1334456 | 1335133 | + | 1334348 | 1335130 | 225 | 261 | ANO48741.1 |
| WP_050855773.1 | 1373831 | 1374163 | + | 1373774 | 1374163 | 110 | 129 | WP_041253394.1 |
| WP_041253205.1 | 1437620 | 1438063 | + | 1437407 | 1438063 | 147 | 218 | WP_065213256.1 |
| WP_050855827.1 | 2158363 | 2158932 | + | 2155312 | 2158932 | 189 | 1206 | ANO47058.1 |
| WP_0411253460.1 | 2161502 | 2161690 | + | 2158994 | 2161690 | 62 | 898 | WP_034708674.1 |
| WP_014165932.1 | 2165644 | 2167200 | - | 2165644 | 2167248 | 518 | 534 | ANO47062.1 |
| WP_041253481.1 | 2289351 | 2289980 | + | 2289273 | 2289980 | 209 | 235 | ANO47171.1 |
| WP_014166036.1 | 2291006 | 2291926 | + | 2290922 | 2291926 | 306 | 334 | WP_063743531.1 |
| WP_014166109.1 | 2384684 | 2385160 | - | 2384684 | 2385196 | 158 | 170 | ANO47258.1 |
| WP_041253501.1 | 2456189 | 2460493 | - | 2456189 | 2460553 | 1434 | 1454 | ANO47801.1 |
| WP_050855795.1 | 2504876 | 2505193 | - | 2504876 | 2505307 | 105 | 143 | WP_060382934.1 |
| WP_041253513.1 | 2532536 | 2532964 | + | 2532185 | 2532964 | 142 | 259 | ANO47738.1 |
| WP_014166306.1 | 2637558 | 2637800 | + | 2637453 | 2637800 | 80 | 115 | WP_065213069.1 |
| WP_014166307.1 | 2637896 | 2638450 | + | 2637836 | 2638450 | 184 | 204 | ANO47633.1 |
| WP_041253585.1 | 2975929 | 2976924 | - | 2975929 | 2977008 | 331 | 359 | ANO48328.1 |
