# Supplementary material for: Comparative Genomics and Transcriptional Analysis of Flavobacterium columnare Strain ATCC 49512
Source: Front Microbiol. 2017 Apr 19;8:588. doi: 10.3389/fmicb.2017.00588 (PMC5395568; doi:10.3389/fmicb.2017.00588)
Supplement: Supplementary Table 8 — F. columnare ATCC 49512 putative non-coding RNAs based on transcriptome analysis. [file Table8.DOCX]

**Supplementary Table 8. *F. columnare* ATCC 49512 putative non-coding RNAs based on transcriptome analysis**

| **EIR start** | **EIR stop** | **Promoter start** | **Promoter stop** | **Strand** |
| --- | --- | --- | --- | --- |
| 404494 | 404685 | 404558 | 404588 | - |
| 772592 | 773083 | 772954 | 772984 | - |
| 1447554 | 1447842 | 1447683 | 1447712 | - |
| 1606649 | 1607364 | 1607231 | 1607260 | - |
| 2159131 | 2159415 | 2159196 | 2159225 | - |
|  |  |  |  |  |
